# The developmental origins of Notch-driven intrahepatic bile duct disorders

**DOI:** 10.1242/dmm.048413

**Published:** 2021-09-22

**Authors:** Anabel Martinez Lyons, Luke Boulter

**Affiliations:** MRC Human Genetics Unit, Institute of Genetics and Cancer, Edinburgh EH4 2XU, UK

**Keywords:** Bile duct, Cholangiocyte, Liver, Notch

## Abstract

The Notch signaling pathway is an evolutionarily conserved mechanism of cell–cell communication that mediates cellular proliferation, cell fate specification, and maintenance of stem and progenitor cell populations. In the vertebrate liver, an absence of Notch signaling results in failure to form bile ducts, a complex tubular network that radiates throughout the liver, which, in healthy individuals, transports bile from the liver into the bowel. Loss of a functional biliary network through congenital malformations during development results in cholestasis and necessitates liver transplantation. Here, we examine to what extent Notch signaling is necessary throughout embryonic life to initiate the proliferation and specification of biliary cells and concentrate on the animal and human models that have been used to define how perturbations in this signaling pathway result in developmental liver disorders.

## Abstract (precis)

**Summary:** In this At a Glance article and poster, we explore the mechanisms of Notch signaling and discuss how the field has concluded that Notch signaling is a central determinant in bile duct formation and growth.

## Introduction

Patterning of the vertebrate body is a complex process that requires establishment of tissue boundaries, promotion and restriction of cellular differentiation, and correct organ morphogenesis. During liver development, bi-potent epithelial progenitor cells known as hepatoblasts become either hepatocytes, which form the metabolic parenchyma of the liver, or cholangiocytes, which compose the epithelium of the bile ducts and aid in the transport of toxic bile away from the liver. Together, human genetic studies and murine, fly and zebrafish models have highlighted Notch signaling as a master regulator of lineage specification during liver development. Furthermore, Notch signaling is becoming increasingly recognized as an important regulator of ductular morphogenesis and patterning, a role that may continue into postnatal and adult life. In this At a Glance article and poster, we summarize the evidence that Notch signaling plays an instructive and essential role in intrahepatic biliary development and disease.

**Figure:**
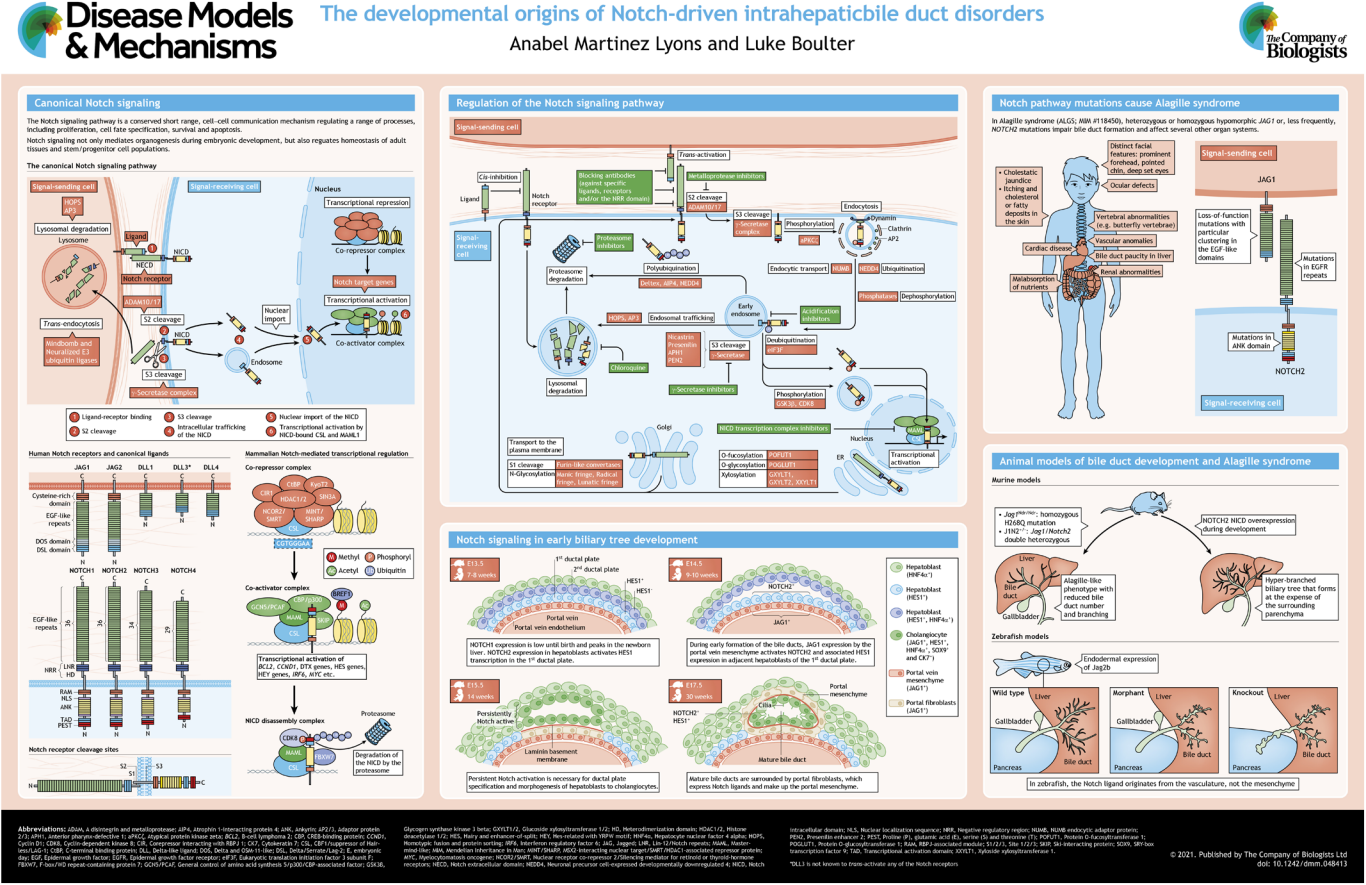

## Overview of the Notch signaling pathway

The Notch signaling pathway is a conserved mechanism of short-range cell–cell communication that mediates a number of essential cellular processes, including proliferation, cell fate specification, cellular survival and apoptosis (Hori et al., 2013; Bray, 2016; Ho and Artavanis-Tsakonas, 2016; Nowell and Radtke, 2017). Aberrations in Notch signaling were first reported in *Drosophila melanogaster*, with the observation and characterization of a dominant notched-wing phenotype resulting from haploinsufficiency of its single *Notch* gene (Dexter, 1914; Morgan, 1917). In the century that followed, the dysregulation and dysfunction of this pathway have been implicated in an increasing number of human diseases (Alagille et al., 1987; Joutel et al., 1996; Penton et al., 2012).

The mammalian Notch system consists of four Notch receptors (Notch1-4), five canonical ligands [Jagged (JAG)1/2, and Delta-like (DLL)1/3/4], and numerous auxiliary factors that regulate and transduce the signal (Artavanis-Tsakonas et al., 1999; Kopan, 2012) (see poster). In canonical Notch signaling, the binding of the Notch extracellular domain (NECD) to the extracellular region of a corresponding ligand on a neighboring cell triggers a conformational change in the receptor that allows for proteolytic cleavage of the receptor at site 2 (S2) by metalloproteinases ADAM10 and ADAM17 (Sapir et al., 2005; Groot and Vooijs, 2012). The NECD and bound ligand are then endocytosed by the signal-sending cell, a process primarily mediated by E3 ubiquitin ligase mindbomb 1 (MIB1) and the ubiquitin-binding adaptor EPSIN proteins (Le Borgne et al., 2005a; Wang and Struhl, 2005). This intermediate state, known as the Notch extracellular truncation (NEXT), then undergoes further proteolytic cleavage at site 3 (S3) by *γ*-secretase (De Strooper et al., 1999), an enzymatic complex that contains presenilin, nicastrin, PEN2 (also called PSENEN) and the APH1 proteins (Fortini, 2002; Bray, 2006; Kopan, 2012). This cleavage releases the Notch intracellular domain (NICD) (Hori et al., 2013), the only direct messenger in the Notch signaling pathway, which then translocates to the nucleus (see poster) (Kopan and Ilagan, 2009). Whether the proteolytic release of the NICD in humans requires endocytic internalization of the receptor prior to S3 cleavage is somewhat debated (Wilkin and Baron, 2005; Fortini and Bilder, 2009; Fürthauer and González-Gaitán, 2009). Evidence from fly studies suggest that *γ*-secretase cleaves the NICD most efficiently following the incorporation of a Notch receptor into early endosomes (Lah and Levey, 2000; Gupta-Rossi et al., 2004; Vaccari et al., 2008; Windler and Bilder, 2010). However, successful S3 cleavage at the plasma membrane has also been demonstrated in mammalian *γ*-30 cells (Chyung et al., 2005). Inside the nucleus, the NICD interacts with DNA-binding transcriptional co-activator proteins C promoter-binding factor 1 (CBF-1)/suppressor of hairless [Su(H)]/Lin-12 and Glp-1 (LAG-1) (CSL; also called RBPJκ and RBPJ) and Mastermind-like (MAML) protein to displace a CSL-bound transcriptional repressor complex (Bray, 2006; Borggrefe and Oswald, 2009). Upon interaction with the NICD, CSL becomes a potent transcriptional activator of target genes, most commonly those belonging to the Hairy and enhancer-of-split (HES) and Hes-related with YRPW motif (HEY) families (Iso et al., 2003; reviewed in Fischer and Gessler, 2007). In mammals, CSL/RBPJκ is essential, and embryos lacking RBPJκ die early in development (Oka et al., 1995). Counterintuitively, the transcription factors encoded by HES and HEY genes typically act as transcriptional repressors (Davis and Turner, 2001; Jones, 2004). In humans, transcriptional repression by HES and HEY genes negatively regulates differentiation, allowing progenitor-like or stem cell populations to remain in an undifferentiated state (Kageyama et al., 2007).

Non-canonical Notch signaling is defined as either dependent or independent of ligand-receptor binding, and does not require nuclear translocation of the NICD (Shawber et al., 1996; D'Souza et al., 2010; Andersen et al., 2012). The best-characterized role of non-canonical Notch signaling is as a negative regulator of the Wnt/β-catenin pathway, which is crucial for the maintenance of progenitor-like/stem cell populations, cell fate specification and proliferation (Hayward et al., 2005; Andersen et al., 2012). Wnt/β-catenin signaling does not occur in developing bile ducts (Cordi et al., 2016), nor does it facilitate bile duct regeneration following injury (Pepe-Mooney et al., 2019; Wilson et al., 2020); however, β-catenin-independent signaling could still play a role in bile duct biology (Okabe et al., 2016). Whether Wnt/β-catenin signaling is actively suppressed by non-canonical Notch signaling during bile duct development remains unclear.

## Central components of the Notch signaling pathway

Notch receptors are cell membrane-spanning multi-domain glycoproteins, the characteristic structure of which is iteratively used from invertebrates to humans (Fleming, 1998; Mizutani et al., 2001). Mammalian Notch receptors anchor to the cell membrane via a single-pass transmembrane region (see poster), flanked by one intracellular and one extracellular domain: the NICD and NECD, respectively (Gordon et al., 2008). The N-terminal NECD of the four human receptors contains between 29 and 36 epidermal growth factor (EGF)-like repeats depending on the homolog (Fleming, 1998), which can bind calcium (Cordle et al., 2008). Downstream of the EGF-like repeats is a negative regulatory region (NRR), composed of three cysteine-rich Lin-12/Notch (LNR) repeats and a hydrophobic heterodimerization domain (Kopan and Ilagan, 2009), which is typically produced by site 1 (S1) proteolytic cleavage by Furin-like convertases during post-translational maturation of the receptor in the Golgi complex (Gordon et al., 2009). The structure of the NRR prohibits ligand-independent activation of the receptor prior to ligand interaction by protecting S2 from metalloproteases (Bray, 2006; Kopan and Ilagan, 2009). Following the NRR is a short transmembrane region and the NICD. The NICD consists of an RBPJκ/CBF1-associated module (RAM) domain (Deregowski et al., 2006) and seven ankyrin (ANK) repeats that are flanked by nuclear localization sequences (NLSs) (Kurooka et al., 1998; Huenniger et al., 2010). These are followed by a transcriptional activation domain (TAD), which is only structurally and functionally conserved in NOTCH1 and NOTCH2 in humans, with a minimally conserved TAD found in NOTCH3 and no TAD in NOTCH4 (Ong et al., 2006). Lastly, the C-terminal PEST domain is required for degradation of the NICD following transcriptional activation (Hori et al., 2013).

Like the Notch receptors, the canonical Delta/Serrate/Lag-2 (DSL) ligands are single-pass transmembrane glycoproteins that have recurrent structures throughout Metazoan evolution (Henderson et al., 1994; Parks et al., 2006; D'Souza et al., 2008) (see poster). *Drosophila* produce two Notch ligands, Delta and Serrate, which have five canonical mammalian orthologs: three belonging to the Delta-like family (DLL1/3/4) and two Serrate homologs, jagged 1 and jagged 2 (JAG1/2) (D'Souza et al., 2010; Hori et al., 2013). DSL ligands contain a variable number of iterative EGF-like repeats and a cysteine-rich DSL domain, which, along with an unstructured N-terminal domain and the first two EGF-like repeats, are essential for Notch receptor binding (Shimizu et al., 1999; Parks et al., 2006). The intracellular regions of DSL ligands lack any obvious sequence homology except that most, but not all, consist of multiple lysine residues and a PSD-95/Dlg/ZO-1 (PDZ) motif (Pintar et al., 2007; D'Souza et al., 2010). More-recent work has described a C2 domain adjacent to this DSL region at the N-terminus of human JAG1 and DLL4. This region mediates phospholipid binding of at least JAG1, JAG2, DLL1 and DLL4, suggesting that phospholipid binding as well as the core function of the DSL ligands are required to confer the Notch signal (Chillakuri et al., 2013). Unexpectedly, a number of pathological mutations in this C2 domain are associated with Alagille Syndrome (ALGS; discussed below), implicating phospholipid–JAG1 interactions in this pleiotropic Notch disorder (Handford et al., 2018).

Mammalian Notch-mediated transcriptional regulation is mechanistically complex, with numerous factors forming co-repressor or co-activator complexes with CSL (see poster). In the presence or absence of NICD, CSL directly binds 5′-CGTGGGAA-3′ motifs in DNA enhancer elements (Tun et al., 1994). In the absence of NICD, CSL recruits and coordinates a number of co-repressor proteins, including CtBP (Chinnadurai, 2002), CtIP (Oswald et al., 2005), CIR-1 (Hsieh et al., 1999), KyoT2 (Taniguchi et al., 1998), NCOR2/SMRT (Kao et al., 1998) and SHARP proteins, the murine homologs of which are the MINT (also called APBA) proteins (Oswald et al., 2002). In mammals, SHARP/MINT proteins are largely considered the most essential transcription factor alongside CSL for suppressing NICD-mediated transcription through the recruitment of other co-repressors (Oswald et al., 2005; Tsuji et al., 2007; Borggrefe and Oswald, 2009) and histone deacetylases (HDACs), including SIN3A and HDAC1 (Nagy et al., 1997; Zhang et al., 2019). CtBP can also bind to histone methyltransferases EHMT2 (also called G9a), GLP (Ueda et al., 2006), LSD1 and CoREST1 to restrict transcriptional machinery components’ access to DNA (You et al., 2001; Shi et al., 2003).

Following nuclear import of the NICD and its interaction with CSL and MAML protein, co-activating factors are recruited to replace the co-repressor complex. These include several histone acetyltransferases (HATs) that act cooperatively, such as CBP/p300 (Oswald et al., 2001; Dancy and Cole, 2015) and PCAF (Wallberg et al., 2002), called Gcn5 in *Drosophila* (Kurooka and Honjo, 2000). Similarly, histone methylation by BRE1 and RTF1 leads to transcriptional upregulation (Bray et al., 2005; Tenney et al., 2006). The only protein identified to date to be integral to the conversion of CSL from a transcriptional repressor to a NICD-bound transcriptional activator is SKIP (Zhou et al., 2000). SKIP interacts with SMRT in the co-repressor complex to recruit HDACs (Kao et al., 1998) and promotes multimerization of NICD prior to its stepwise assembly with MAML protein and CSL to form the mature co-activator complex (Vasquez-Del Carpio et al., 2011), thereby bridging the functionality of the co-repressor and co-activator complexes.

Lastly, degradation of the NICD and turnover of the co-activator complex are fundamental to the tight spatiotemporal control of Notch signaling *in vivo*. For this, MAML protein recruits the cyclin C/cyclin-dependent kinase-8 (CycC/CDK8) complex to phosphorylate the NICD (Fryer et al., 2004). Phospho-NICD is recognized by the E3 ubiquitin ligase F-box and WD repeat domain-containing protein 7 (FBXW7), which poly-ubiquitylates the NICD, targeting it for proteasomal degradation (Gupta-Rossi et al., 2001; Borggrefe and Oswald, 2009).

## Regulation of the Notch signaling pathway

Given that only one direct messenger (NICD) is produced per signaling molecule (Notch receptor), and the fact that there is no enzymatic means of signal amplification in the pathway, it is somewhat surprising that Notch signaling can mount such diverse and cell type-specific outcomes. To achieve this exquisite level of precision, a number of accessory proteins modify and regulate the Notch signal, which is summarized in the ‘Regulation of the Notch signaling pathway’ panel in the poster.

## Gene dosage

The 1:1 stoichiometric relationship between Notch receptor and NICD suggests that gene dosage plays a crucial role in generating appropriate biological outcomes (Andersson and Lendahl, 2014). Indeed, in *Drosophila*, both haploinsufficiency and an additional copy of its single *Notch* gene result in abnormal morphogenic phenotypes (Lyman and Yedvobnick, 1995; Fanto and Mlodzik, 1999). The *Notch* gene is located on the *Drosophila* X-chromosome, so its heterozygous deletion results in embryonic lethality (Johnson-Schlitz and Lim, 1987). The sensitivity of the mammalian Notch system to gene dosage is most strikingly demonstrated by murine knockout models for *Notch1* (Swiatek et al., 1994), *Notch2* (Hamada et al., 1999), *Dll1* (De Angelis et al., 1997), *Dll4* (Gale et al., 2004) and *Jag1* (Xue et al., 1999), which each present with mid-gestational embryonic lethality. Additionally, *Jag2* homozygous-null mutant mice die soon after birth from cleft palate (Jiang et al., 1998), and although *Notch3-*deficient animals are postnatally viable, they display multiple developmental defects (Domenga et al., 2004; Belin De Chantemèle et al., 2008). Curiously, *Notch4-*deficient mice are both viable and fertile with no obvious aberrant phenotypes, suggesting possible functional redundancy for this receptor during development and postnatal life (Krebs et al., 2000). In humans, haploinsufficiency of *NOTCH2* and *JAG1* cause ALGS, an inherited multisystemic developmental disorder that presents in the liver with loss of the bile ducts (Alagille et al., 1987; Gilbert et al., 2019). Lastly, both gain- and loss-of-function Notch mutations have been implicated in cancer (Park et al., 2006; Roy et al., 2007; Mazzone et al., 2010; Schmitz et al., 2018). In colorectal cancer, for example, *NOTCH1* copy number gain is an important indicator of disease progression and is positively correlated with poor prognosis (Arcaroli et al., 2016), and mice with sporadic, low-frequency loss of NOTCH1 protein expression develop widespread vascular tumors (Liu et al., 2011), highlighting roles for this receptor in both neoplastic transformation as well as tumor suppression, depending on the biological context.

## Regulation in *cis* and in *trans*

Notch receptor-ligand binding typically refers to the *trans*-interaction between a Notch receptor on one cell's surface and a cognate DSL ligand expressed on an opposing or adjacent cell (see poster). The importance of *cis*-interactions between Notch receptors and ligands on the same cell has become a well-defined Notch paradigm over the past decade (reviewed by Del Álamo et al., 2011; Negri et al., 2019). *Cis*-inhibition of Notch receptors by DSL ligands has historically been reported to downregulate Notch signaling (Fehon et al., 1990; Pérez et al., 2005; Matsuda and Chitnis, 2009; Fiuza et al., 2010). In early *Drosophila* experiments, Notch and Delta interact in co-clusters via their extracellular domains (Fehon et al., 1990), and structure–function studies later revealed that the receptor-binding domain of Serrate was responsible for *cis*-inhibition of Notch when expressed on the same cell membrane (Glittenberg et al., 2006). In fact, DLL3 may act exclusively as a *cis*-inhibitor of Notch signaling in mammals, as it is incapable of activating Notch receptors in *trans* (Ladi et al., 2005). Notably, introduction of a *Dll3* expression cassette into the murine *Dll1* locus revealed divergent functions for these ligands, with DLL3 promoting an inhibitory effect and DLL1 promoting an activating effect on Notch signaling (Geffers et al., 2007). Curiously, DLL3 does not readily localize to the plasma membrane, but instead exists almost entirely intracellularly (Geffers et al., 2007). Intracellular interactions of Delta and Serrate with Notch in *Drosophila* prevent Notch receptor from reaching the cell surface (Sakamoto et al., 2002), and intracellular *cis*-interaction between NOTCH1 and JAG1 in mice blocks *trans*-activation of Notch receptors during angiogenesis and pancreatic development (Benedito et al., 2009; Golson et al., 2009). Recent work showed that *cis*-activation of Notch signaling, i.e. from interactions on the same cell membrane, occurs between several DSL ligands (DLL1, DLL4 and JAG1) and Notch receptors (NOTCH1 and NOTCH2) (Nandagopal et al., 2019), although the biological significance of these interactions remains unclear.

## Post-translational processing of the Notch receptors

Perhaps the most crucial means of controlling Notch signaling *in vivo* is the post-translational processing of Notch receptors and ligands, which regulates their maturation, binding avidity, endocytic trafficking and degradation (see poster). Notch receptors typically undergo several different glycan modifications (reviewed in Urata and Takeuchi, 2020). In the endoplasmic reticulum (ER), *O*-fucose can be added to EGF repeats of the NECD by POFUT1 (known as O-fut1 in *Drosophila*) (Okajima and Irvine, 2002), and *O*-glucose can be added by POGLUT1 (known as Rumi in *Drosophila*) (Acar et al., 2008; Fernandez-Valdivia et al., 2011). *O*-Fucosylation is not essential for Notch signal transduction (Okajima et al., 2008; Vodovar and Schweisguth, 2008); however, it is necessary for later glycosylation by Fringe proteins, which in mammals include Manic fringe, Lunatic fringe and Radical fringe (Kakuda and Haltiwanger, 2017). These are β1-3*N*-acetylglucosaminyl transferases that extend *O*-fucose by adding *N*-acetylglucosamine (GlcNAc) (Panin et al., 1997). In *Drosophila*, Fringe modifications increase the interaction affinity between Notch and Delta and reduce the affinity between Notch and Serrate (Brückner et al., 2000; Lei et al., 2003). Similarly, in mice, glycosylation potentiates NOTCH1 interactions with DLL1 and reduces its responsiveness to JAG1 (Hicks et al., 2000; Kato et al., 2010). Loss of *Pofut1* in mice causes embryonic lethality, most likely due to a significant loss of Notch signaling during embryogenesis (Shi and Stanley, 2003). Besides its role in *O*-fucosylation within the ER, *Drosophila* O-fut1 acts as a chaperone in the folding of nascent Notch polypeptides (Okajima et al., 2005), and may also coordinate endocytic trafficking and turnover of Notch receptors at the cell surface (Sasamura et al., 2007). Like POFUT1, POGLUT1 is an essential protein in mice (Fernandez-Valdivia et al., 2011). Analysis of protein extracts from various tissues of *Rumi*-mutant flies and mouse cell lines that had undergone RNA interference (RNAi)-mediated knockdown of *Poglut1* revealed significantly reduced Notch receptor proteolysis, particularly at the S2 cleavage site (Acar et al., 2008; Fernandez-Valdivia et al., 2011). As proteolysis requires conformational changes in the NECD, *O*-glucosylation may couple ligand binding to requisite conformational changes necessary for proteolysis and Notch activation (Rana and Haltiwanger, 2011). Lastly, *O-N*-GlcNAc can be added to EGF repeats of the NECD of human Notch receptors by the ER-localizing protein EOGT1 (Matsuura et al., 2008; Sakaidani et al., 2012; Varshney and Stanley, 2017). Recent work has demonstrated that EOGT1 promotes the binding of NOTCH1 to DLL1 and DLL4, but not to JAG1 (Sawaguchi et al., 2017). Therefore, addition of *O-N*-GlcNAc by EOGT1 may inform ligand-binding affinity of Notch receptors in a similar manner to *O*-fucosylation.

In mammals, Notch receptors may undergo proteolytic processing prior to their presentation at the plasma membrane (see poster). NOTCH1 is well documented to be cleaved at S1 by Furin or Furin-like convertases (Logeat et al., 1998; Gordon et al., 2009). S1 cleavage produces a non-covalently linked heterodimer that predisposes NOTCH1 to proteolytic *trans*-activation (Logeat et al., 1998). However, Furin cleavage is not a requirement for Notch signaling transduction in *Drosophila* (Kidd and Lieber, 2002), nor is it essential for cell surface trafficking or functional activity of mammalian NOTCH1 and NOTCH2 (Gordon et al., 2009). Instead, S1 cleavage may be a regulatory process that enhances the cell surface expression of Notch receptors, rather than an intrinsic step of Notch receptor maturation and functionality. A novel enhancer of S1 cleavage, CRIPTO1 (also called TDGF1), binds to uncleaved Notch receptors to recruit Furin, as well as factors that drive endocytic trafficking to the plasma membrane (Blanchet et al., 2008; Watanabe et al., 2009). *Cripto1*-deficient mouse and human embryonal carcinoma cells showed an intracellular accumulation of non-Furin-cleaved NOTCH1, as well as decreased presence and activation of the receptor at the cell surface (Watanabe et al., 2009). Whether there are other positive regulators of S1 cleavage remains to be determined.

Phosphorylation is a crucial regulatory process in the intracellular routing, stability and turnover of Notch receptors, and is carried out by at least three different kinases: atypical protein kinase Cζ (aPKCζ) (Sjöqvist et al., 2014), glycogen synthase kinase 3β (GSK3β) (Foltz et al., 2002; Espinosa et al., 2003) and CDK8 (Fryer et al., 2004). Following S2 proteolysis, aPKCζ phosphorylates S2-cleaved, membrane-tethered Notch receptors and promotes their endosomal internalization (Sjöqvist et al., 2014). In the absence of ligand binding, aPKCζ facilitates internalization of the full-length Notch receptor, promoting its turnover through interaction with endosomal sorting and ubiquitylating proteins (Sjöqvist et al., 2014). In this way, aPKCζ behaves as both a positive and negative regulator of Notch signaling. GSK3β is a component of the Wnt/Wingless signaling pathway (Wu and Pan, 2010), and stabilizes and prevents degradation of the NICD when en route to the nucleus (Foltz et al., 2002). GSK3β inhibition increases cell surface expression and activation of NOTCH1, indicating a potential role for GSK3β in negatively regulating Notch signaling in homeostatic conditions (Zheng and Conner, 2018). Lastly, CDK8 acts predominantly in the downregulation of Notch signaling by phosphorylating the NICD in the nucleus, allowing for its ubiquitylation and subsequent degradation by the proteasome (Fryer et al., 2004).

Ubiquitylation of lysine residues within the intracellular domains of Notch receptors and DSL ligands is conserved across species (Heuss et al., 2008). The addition of one or more ubiquitin monomers to a single lysine residue, or to several different lysine residues, directly influences the cell-surface expression, internalization, endosomal routing and degradation of Notch signaling components (Yamamoto et al., 2010; Le Bras et al., 2011; Moretti and Brou, 2013). Monoubiquitylation by RING-type E3 ubiquitin ligases Neuralized [*Neur1* and *Neur2* (also called *Neurl1a* and *Neurl1b*) in mammals] and Mindbomb (*Mib1* and *Mib2* in mammals) promotes the activation, as well as endocytosis, of DSL ligands following their interaction with a Notch receptor (Le Borgne et al., 2005b; Song et al., 2006). Another type of RING-type E3 ubiquitin ligase, Deltex, is an important positive regulator of ligand-independent Notch activation in the non-canonical Notch signaling pathway. In *Drosophila*, Deltex promotes the late-endosomal activation of Notch, and, in mammals, Deltex proteins promote the late-endosomal activation of various mammalian Notch receptor homologs (Matsuno et al., 1998; Ramain et al., 2001; Hori et al., 2004). In opposition to these signaling activators, NUMB, an endocytic adaptor protein (McGill et al., 2009), indirectly suppresses Notch signaling by recruiting ubiquitin ligase AIP4, called Suppressor of deltex [Su(dx)] in *Drosophila* and ITCH in mice and other vertebrates (McGill and McGlade, 2003). AIP4 negatively regulates Notch signaling in a ligand-independent manner by polyubiquitylating Deltex protein and the intact NICD of full-length Notch receptors (Qiu et al., 2000; Chastagner et al., 2006). NUMB differentially affects the four mammalian Notch homologs, which may increase the diversity of possible Notch signaling outcomes within various cell populations (Beres et al., 2011). Additionally, humans express at least six differentially spliced NUMB isoforms (NUMB1-6) (Andersson et al., 2011). *Drosophila* Numb also recruits Nedd4, which regulates the endocytic internalization and ligand-independent activation of Notch receptors (Sakata et al., 2004; Wilkin et al., 2004). Finally, eIF3F, a subunit of the translation initiation factor eIF3, is a deubiquitylase that targets activated forms of Notch, i.e. following S2 cleavage (Zhou et al., 2008).

## Notch transcriptional outcome depends on ligand and tissue type

The diversity of Notch signaling responses varies depending on the cell or tissue type and stage of life (reviewed in Andersson et al., 2011). For example, Delta/DLL and Serrate/JAG ligands generate distinct transcriptional responses in neighboring cells within the same tissue (Kooh et al., 1993; Lewis, 1998; Artavanis-Tsakonas et al., 1999; Zecchin et al., 2007; Lake et al., 2009). In the liver, key molecular determinants of bile duct fate are upregulated by Notch signaling, including *SOX9*, *TGFB* and *HNF1B* (Geisler and Strazzabosco, 2015). Below, we examine in more detail the evidence that Notch signaling and its regulation play a pivotal role in biliary development.

## Notch signaling in liver development

In the embryonic liver, bile ducts arise from a transient structure known as the ductal plate (see ‘Notch signaling in early biliary tree development’ panel in the poster). The ductal plate forms from liver progenitor cells called hepatoblasts that undergo complex morphogenic rearrangements to form a functional tubular network. In mammals, bile ducts develop at different rates across the embryonic liver, with the large ducts close to the hilum forming prior to the smaller intrahepatic ducts at the periphery (Antoniou et al., 2009). The adult biliary tree is a continuous ductular network; however, during development, multiple regions of the ductal plate form separate pseudo-ductular structures that connect as they grow to create a contiguous structure with a continuous lumen (Tanimizu et al., 2016).

There are a number of congenital diseases of the bile ducts, which typically arise from malformation of the ductal plate during embryonic development (see poster). One such example is ALGS (Alagille et al., 1975; Li et al., 1997; Oda et al., 1997), in which patients are born largely lacking intrahepatic bile ducts (Alagille et al., 1987). Mutations in *JAG1* account for ∼94% of ALGS cases, and causal hypomorphic mutations in *NOTCH2* (McDaniell et al., 2006) are thought to occur in ∼2.5% of cases. Interestingly, the remaining 3.2% remain genetically uncharacterized (Gilbert et al., 2019).

A number of model organisms have been developed in an attempt to recapitulate the bile duct paucity phenotype seen in ALGS patients (see ‘Animal models of bile duct development and Alagille syndrome’ panel in the poster). Early work in zebrafish showed that endodermal expression of *jag2b*, a homolog of mammalian Jagged, is essential for the formation of ducts in the liver (Lorent et al., 2004; Zhang et al., 2017), and that its loss results in a failure to form bile ducts. Interestingly, in this model, Notch signaling is dispensable for the formation of the hepatocyte-rich parenchyma (Lorent et al., 2010). Furthermore, mice that are doubly heterozygous for loss of *Jag1* and hypomorphic *Notch2* exhibit a significant paucity of bile ducts (McCright et al., 2002). However, as *Jag1* heterozygous mutant mice already have an ALGS phenotype it is difficult to ascertain the exact effect of hypomorphic *Notch2* in this system. These studies, however, fail to conclude whether ductular agenesis is due to reduced specification of ductular cells from hepatoblasts, or whether aberrant Notch signaling inhibits normal ductular formation and morphogenesis.

Notch signaling typically requires the interaction of ligands and receptors on adjacent cells; this cell–cell signaling results in signal directionality, where one cell influences the fate of the other by repressing or inducing specific lateral inhibition and lateral induction transcriptional programs, respectively (Sjöqvist and Andersson, 2019). Deletion of *Jag1* during murine liver development in the portal mesenchyme specifically, but not in endothelial cells, is sufficient to reduce the specification of cholangiocytes from hepatoblasts (Hofmann et al., 2010). The resulting livers have a bile duct paucity phenotype that closely resembles that of ALGS patients (Hofmann et al., 2010), suggesting that Notch signaling from the portal mesenchyme spatially constrains the formation of the ductal plate close to the portal vein. This lies in contrast to the equivalent process in zebrafish, in which Jag-type ligands are restricted to the vascular endothelial cells and not the adjacent mesenchyme (Zhang et al., 2017). In addition, the genetic deletion of *Notch2* (McCright et al., 2006; Geisler et al., 2008; Falix et al., 2014) or of the DNA-binding co-transcriptional activator gene *Csl* (also called *Rbpj*/*CBF1*/*RBPJκ* in mice) in the developing murine liver results in bile duct agenesis (Sparks et al., 2011). Despite the structural and functional similarities between NOTCH1 and NOTCH2, deletion of *Notch1* in bile duct development does not affect bile duct formation (Geisler et al., 2008).

Genetic evidence from ALGS patients and animal models have proven that a loss of NOTCH2 is sufficient for ductular paucity (McCright et al., 2002; Lozier et al., 2008; Andersson et al., 2018). However, overexpression of the NOTCH2 intracellular domain during liver development results in the formation of many more ductular structures than normal, at the expense of the surrounding parenchyma, highlighting NOTCH2 as a central fate regulator in the ductular lineage (Dill et al., 2013). Collectively, these data implicate Notch signaling in the early specification of the biliary lineage, but not necessarily in subsequent morphogenesis.

As detailed above, multiple proteins modulate the length and strength of the Notch signal. In particular, the mammalian Fringe proteins interact with a heterozygous mutant of *Jag1* (Ryan et al., 2008). Here, deletion of Fringe proteins from the liver results in the postnatal expansion of the biliary tree, suggesting that glycosylation of the Notch receptor NECD suppresses Notch activity during liver development. Interestingly, in this system, not all Fringe proteins contribute to ductular growth in the same way: whereas loss of Lunatic fringe and Radical fringe promote a robust expansion of the biliary tree, loss of Manic fringe results in a more subtle phenotype, suggesting that different Fringe proteins may modify Notch receptors in bile duct development to fine-tune the level of signaling activity. Similarly, deletion of one copy of *Poglut1* (Fernandez-Valdivia et al., 2011; Thakurdas et al., 2016) increases JAG1 expression, thereby overcoming the ALGS phenotype induced by *Jag1* haploinsufficiency. Together, these data highlight how fine-tuning of the Notch pathway is necessary for the development of a functional biliary tree that is also patterned correctly.

Classical genetic studies and animal models have demonstrated that *Jag1* and *Notch2* are essential for bile duct development (Lorent et al., 2004; Geisler et al., 2008; Gilbert et al., 2019). Activation of the canonical Notch signaling pathway affects the expression of the biliary-enriched transcription factor *Hnf1b*, which is essential for biliary specification and differentiation (Tanimizu and Miyajima, 2004). Furthermore, the transcription factor SOX9 is essential for bile duct formation (Poncy et al., 2015), and modifies JAG1 phenotypes in murine liver, whereby haploinsufficiency of *Sox9* cooperates with *Jag1* heterozygosity and worsens the bile duct paucity (Poncy et al., 2015). This relationship between SOX9 and Notch signaling has also been identified in ALGS patients, in which *SOX9* expression levels are inversely correlated with disease severity. These interactions suggest that SOX9 expression directly influences Notch signaling, and, indeed, *Notch2* is a transcriptional target of SOX9 (Adams et al., 2020). Interestingly, increasing the ectopic expression of SOX9 during liver development rescues the JAG1 pathogenic phenotype by promoting higher levels of *Notch2* expression, thereby compensating for the loss of Notch signaling in this system (Thakurdas et al., 2016; Adams et al., 2020). *Sox9* is also a Notch target gene in bile duct development (Russell et al., 2019), suggesting that a Notch–SOX9 positive-feedback loop could be essential for establishing the specification of bile ducts.

Notch signaling is well established to be necessary for the specification and differentiation of hepatoblasts into cholangiocytes (Tanimizu and Miyajima, 2004; Antoniou et al., 2009; Zong et al., 2009), but emerging evidence implicates this pathway in bile duct morphogenesis (Lozier et al., 2008; Fiorotto et al., 2013). In a zebrafish model of bile duct development in which Notch signaling activity was reported by the expression of enhanced green fluorescent protein (eGFP), Notch signaling was found to promote morphogenic changes necessary to form a continuous ductular network (Lorent et al., 2010). In mammals, there is also evidence that Notch signaling continues to be important in ductular patterning beyond specification of the bile ducts from hepatoblasts. The Nodder mouse, which contains a homozygous H268Q mutation in *Jag1*, has broad systemic phenotypes similar to patients with ALGS (Hansson et al., 2010). JAG1^H268Q^ retains its ability to bind NOTCH2, but is unable to bind NOTCH1, and has a diminished capacity to bind NOTCH3 (Hansson et al., 2010). In this model, the differentiation of biliary epithelial cells is impaired, and ductular formation is delayed. In addition, bile ducts of Nodder mice exhibit differences in structural stability where the distribution of proteins that are typically localized to the apical surface of cells is lost, indicating that Notch signaling either directly or indirectly contributes to or maintenances apical-basal polarity in cholangiocytes (Andersson et al., 2018).

## Conclusions

Notch signaling is clearly central to liver development and patterning of the vertebrate biliary system. Failure to form a functional biliary network remains compatible with life, as demonstrated by patients with ALGS and animal models with a bile duct agenesis phenotype (Andersson et al., 2018; Elkhoury et al., 2019). When ducts fail to form in the absence of Notch signaling (Walter et al., 2014), postnatal murine development can correct for their absence or atrophy through a TGFβ-dependent mechanism (Schaub et al., 2018). Whether this mechanism is widely applicable to all vertebrates, and whether it buffers the effects of small biliary malformations in development, remains unclear.

Further work is required to unpick the inter-relationships between Notch signaling and other signaling pathways in ductular formation and morphogenesis. With a growing number of cellular and *in vivo* model systems, late-developmental events may be better understood and the temporal role of Notch signaling investigated to give us a more comprehensive view of Notch as a master regulator of bile duct development. Understanding ductular formation in the embryo directly informs our understanding of the processes in adult ductular regeneration and cancer, contexts in which re-activation of the Notch signaling pathway is thought to occur. In these pathophysiological contexts, pharmacological modulation of Notch signaling could represent an attractive candidate as a pro-regenerative or anti-cancer therapy and should be developed for patient benefit.

## Poster

Poster
