# Supplementary figures and images for: The developmental origins of Notch-driven intrahepatic bile duct disorders
Source: Dis Model Mech. 2021 Sep 22;14(9):dmm048413. doi: 10.1242/dmm.048413 (PMC8480193; doi:10.1242/dmm.048413)

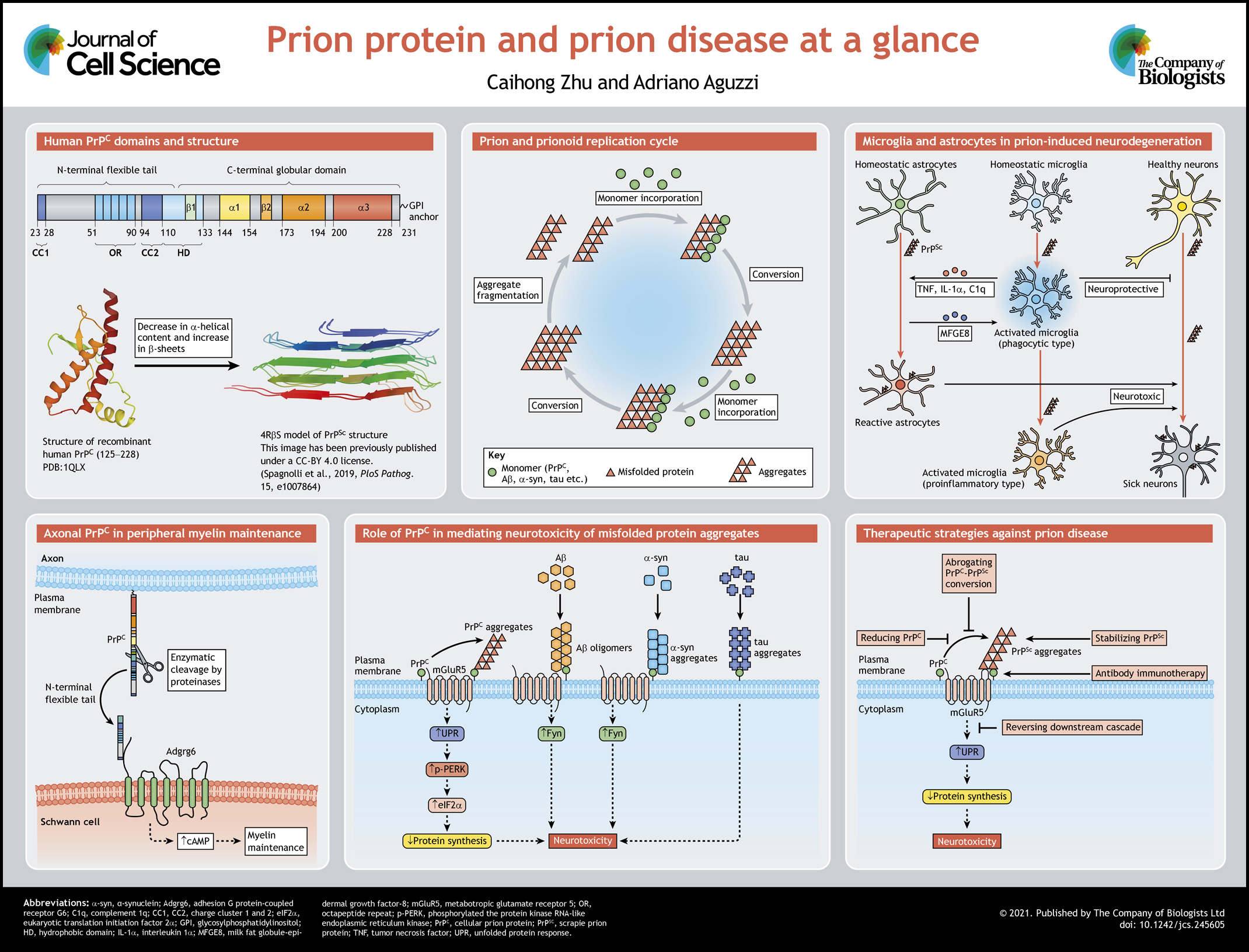

Supplement: Poster [file dmm-14-048413-s1.jpg]
